# Large-area mapping of active cropland and short-term fallows in smallholder landscapes using PlanetScope data

**DOI:** 10.1016/j.jag.2022.102937

**Published:** 2022-08

**Authors:** Philippe Rufin, Adia Bey, Michelle Picoli, Patrick Meyfroidt

**Affiliations:** a Earth and Life Institute, UCLouvain, 1348 Louvain-la-Neuve, Belgium; b Geography Department, Humboldt-Universität zu Berlin, 10117 Berlin, Germany; c F.R.S.-FNRS, 1000 Brussels, Belgium

**Keywords:** Mozambique, Sub-Saharan Africa, Shifting cultivation, Agriculture, Land use, Sentinel-2, Coregistration, Time series, Google Earth Engine

## Abstract

Cropland mapping in smallholder landscapes is challenged by complex and fragmented landscapes, labor-intensive and unmechanized land management causing high within-field variability, rapid dynamics in shifting cultivation systems, and substantial proportions of short-term fallows. To overcome these challenges, we here present a large-area mapping framework to identify active cropland and short-term fallows in smallholder landscapes for the 2020/2021 growing season at 4.77 m spatial resolution. Our study focuses on Northern Mozambique, an area comprising 381,698 km^2^. The approach is based on Google Earth Engine and time series of PlanetScope mosaics made openly available through Norwaýs International Climate and Forest Initiative (NICFI) data program. We conducted multi-temporal coregistration of the PlanetScope data using seasonal Sentinel-2 base images and derived consistent and gap-free seasonal time series metrics to classify active cropland and short-term fallows. An iterative active learning framework based on Random Forest class probabilities was used for training rare classes and uncertain regions. The map was accurate (area-adjusted overall accuracy 88.6% ± 1.5%), with the main error type being the commission of active cropland. Error-adjusted area estimates of active cropland extent (61,799.5 km^2^ ± 4,252.5 km^2^) revealed that existing global and regional land cover products tend to under-, or over-estimate active cropland extent, respectively. Short-term fallows occupied 28.9% of the cropland in our reference sample (13% of the mapped cropland), with consolidated agricultural regions showing the highest shares of short-term fallows. Our approach relies on openly available PlanetScope data and cloud-based processing in Google Earth Engine, which minimizes financial constraints and maximizes replicability of the methods. All code and maps were made available for further use.

## Highlights

• PlanetScope mosaics used for mapping smallholder agriculture in Mozambique.
• Multi-temporal coregistration improved with seasonal Sentinel-2 reference images.
• Active learning based on Random Forest class probabilities.
• Accurate map of active cropland and short-term fallows at 4.77 m resolution.

## 1 Introduction

Smallholder crop production is a key livelihood in many tropical regions and has a substantial relevance for food security. Simultaneously, agricultural expansion has been identified as a principal driver of land use change, including the clearing of forests, savannas, and other ecosystems. Timely and spatially detailed maps of cropland extent are essential for assessing the productivity of agricultural land by evaluating crop types and yields (Jin et al., 2019, Lambert et al., 2018), particularly in regions vulnerable to extreme climatic events (Nakalembe et al., 2021). Likewise, data on the spatial distribution of croplands are essential for monitoring land use change (Bey et al., 2020) and associated carbon emissions (Sy et al., 2019). Earth observation technologies offer great potential for providing timely wall-to-wall cropland maps. However, cropland mapping is not fully operational in complex smallholder landscapes, in contrast to consolidated agricultural systems (d’Andrimont et al., 2021, Song et al., 2021). Consequently, information on cropland distribution is either scarce or does not meet the particularly high requirements for timeliness, spatial resolution, and thematic detail for smallholder-dominated agricultural landscapes.

Key challenges for smallholder cropland mapping are 1) spatial fragmentation, 2) within-field heterogeneity, 3) rapid dynamics, and 4) fallowing as an integral component of the agricultural system. These challenges are particularly complicating mapping in vast swaths of Sub-Saharan Africa (SSA), where agricultural landscapes are highly fragmented and the majority of crop fields are smaller than 0.64 ha (Lesiv et al., 2019). Furthermore, land management in smallholder landscapes of SSA is unmechanized, labor-intensive, and nearly free of chemical inputs. Burning, manual land clearing and preparation, heterogenous management skills and labor inputs, as well as the presence of shading trees and shelters, lead to high within-field heterogeneity in terms of vegetation types and cover density. Smallholder agriculture in SSA often undergoes shifting cultivation, with frequent rotations between active cropland and short-term fallows, resulting in dynamic, mosaic landscapes with substantial fractions of fallow land in various stages and conditions (Tong et al., 2020). To be the most useful, monitoring thus requires timely maps of high granularity allowing for untangling actively used cropland from fallows, in order to precisely assess the land area used for agricultural production, as well as the overall land footprint of agriculture in a landscape.

Approaches based on openly available Landsat and Sentinel-2 (S2) (30–10 m) imagery, yield timely and high-resolution maps of cropland extent at the global (Karra et al., 2021 - 2021, Zanaga et al., 2021), national (Estes et al., 2021, Kerner et al., 2020) and sub-national level (Bey et al., 2020, Ibrahim et al., 2021). In the most fragmented landscapes of SSA, however, the spatial resolution of these data still hampers the accurate identification of cropland, as indicated in spatially explicit accuracy assessments (Tsendbazar et al., 2021) and regional comparisons of mapped cropland area (Lambert et al., 2016, Wei et al., 2020). These spatial complexities highlight the need for approaches based on image data with a spatial resolution below 10 m. Furthermore, the high prevalence of cloud cover in the growing seasons of tropical and sub-tropical regions often limits data availability for sensors with near-weekly revisit intervals. Pre-processing approaches that aggregate images across multiple years may help to mitigate these limitations, but these may obfuscate the spatio-temporal patterns of cropland distribution due to the rapid changes. Lastly, fallows are often included in generic cropland definitions of existing map products (Nabil et al., 2021), likely due to limitations in spatial resolution and minimum mapping units, spectral-temporal similarities between fallows and other land covers, and challenges for reference data collection. Consequently, knowledge about the shares and spatial distribution of fallow land in smallholder systems of SSA is currently scarce.

The PlanetScope satellite constellation provides visible to near infrared imagery at 3–5 m resolution at near-daily intervals (Roy et al., 2021) and thereby offers novel opportunities to overcome persisting challenges for cropland mapping in smallholder agriculture. Recent studies propose enhanced pre-processing routines to improve the quality of PlanetScope data (Scheffler et al., 2017), and demonstrate the use of these data for assessments of vegetation phenology or water use (Aragon et al., 2021, Cheng et al., 2020). These applications are promising, but mostly have experimental character, focusing on local to regional scale study sites, partly because PlanetScope image time series across large regions are costly to obtain. Due to constraints in financial and technical resources in many smallholder-dominated countries, the computational infrastructure, tools, and data and to produce such maps need to be accessible at a low cost. In this regard, Norwaýs International Climate and Forest Initiative (NICFI) launched the NICFI data program (Planet Labs Inc., 2020b), releasing 4.77 m 4-band PlanetScope mosaics across the world́s tropics at monthly intervals from September 2020 onwards. These data were made available within Google Earth Engine cloud computing platform (Gorelick et al., 2017), which offers novel opportunities for large-area mapping at high granularity. To date, however, the potential of these data for characterizing complex smallholder landscapes has not been explored.

We here advance the current state-of-the-art by presenting a framework for mapping active and fallow cropland across a large region using openly available PlanetScope time series in conjunction with S2 to enhance multi-temporal coregistration consistency. We focus our analysis on Northern Mozambique because the region is particularly heterogeneous and dynamic, and because recent and accurate information on active and fallow cropland extent is scarce. Our objectives are to:

1) Present a framework based on openly available PlanetScope time series for large area land cover mapping in Google Earth Engine.
2) Map and assess the distribution of active and short-term fallow cropland in Northern Mozambique for the cropping season 2020/2021.
3) Assess differences in cropland extent derived from regional and global land cover products with the presented map and unbiased area estimates.

## 2 Data & methods

### 2.1 Study area

**Fig. 1:**
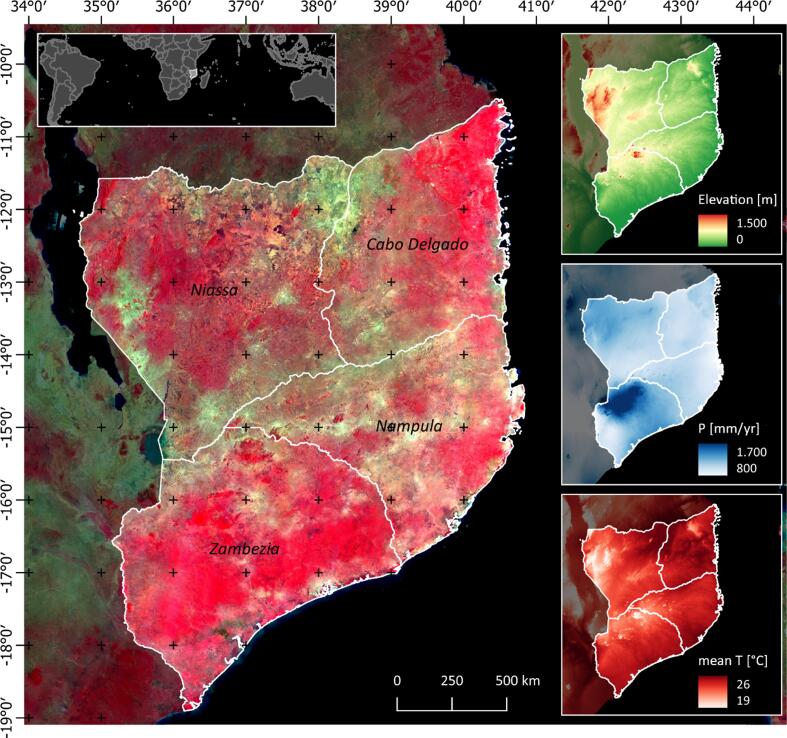
Study region in Northern Mozambique. PlanetScope Mosaic for September 2020 in false-color infrared (R: near infrared, G: red, B: green). Insets show elevation, total annual precipitation, and mean temperature from BioClim data (Fick and Hijmans, 2017). (For interpretation of the references to color in this figure legend, the reader is referred to the web version of this article.)

Our study area comprises four provinces in Northern Mozambique (Cabo Delgado, Nampula, Niassa, and Zambezia), excluding lake Malawi, encompassing a total area of 381,698 km^2^ (Fig. 1). The region is dominated by Eastern Miombo woodlands covering vast parts of the western and central study region. Total annual precipitation ranges between 800 and 1700 mm/year, with the highest levels in the northern parts of Zambezia province and the Lichinga plateau in the western parts of Niassa province. Ferrasols and Lixisols are the dominant soil types. The west-to-east elevation gradient drops from 1,500 m on the Lichinga plateau towards the eastern coast with some local topographical features. Agriculture in the region is dominated by low-intensity smallholder agriculture with high labor demands, little to no fertilizer or pesticide inputs, and frequent fallow rotations (Leonardo et al., 2018). Following a vivid land use history, rapid land change dynamics occurred through smallholder expansion and large-scale agricultural investments in the post-2000 era, particularly surrounding the Nacala corridor, which links the major inland production regions with the Nacala harbor at the coast (Bey et al., 2020, Kronenburg García et al., 2021).

### 2.2 Workflow

**Fig. 2:**
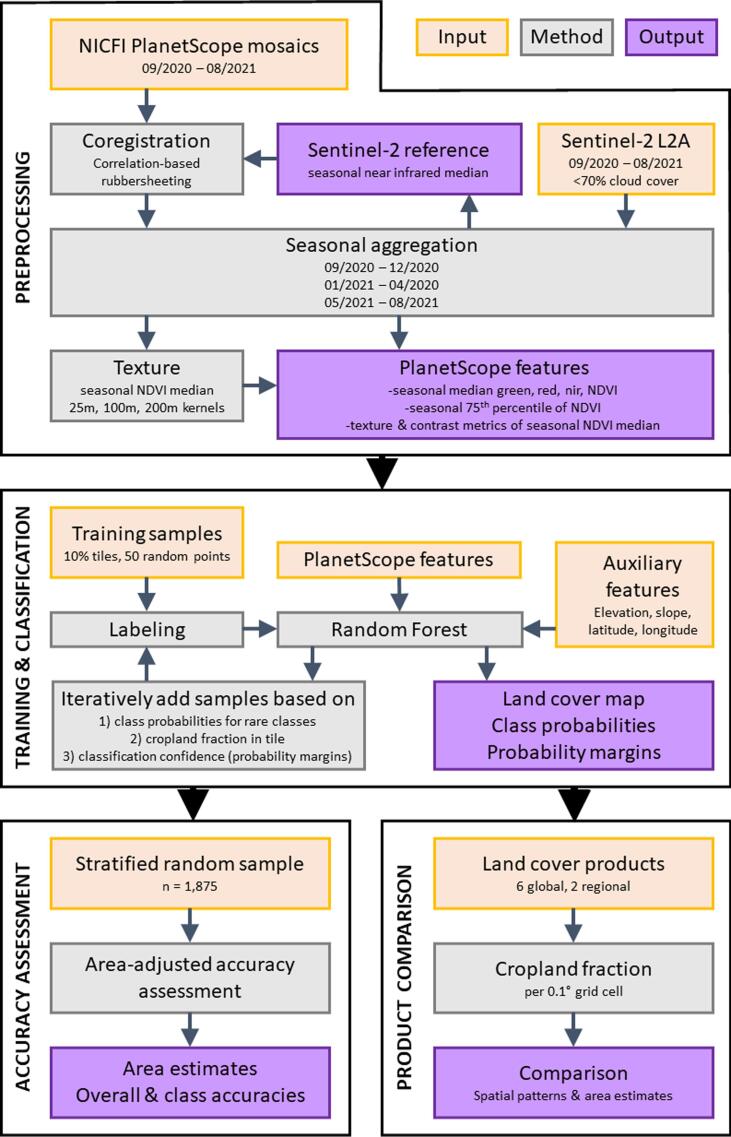
Key workflow elements.

The workflow of our study involves 1) a pre-processing framework to create consistent seasonal input features from the PlanetScope surface reflectance mosaics provided through Level 1 access of the NICFI data program (hereafter PlanetScope mosaics), 2) an iterative active learning scheme for training data collection, model parametrization, and classification, 3) an area-adjusted accuracy assessment and unbiased area estimation, and 4) a comparison of cropland extent and distribution mapped here with regional and global land cover products (Fig. 2). All processing steps to create the map were conducted using the Google Earth Engine Python Application Programming Interface (API), post-processing was performed on local machines with Python and R, and reference data were labeled within QGIS.

### 2.3 Data & pre-processing

We identified multitemporal misregistration in the PlanetScope mosaic time series. We mitigated these errors by matching the geometry of the PlanetScope mosaics for September 2020 through August 2021 (Planet Labs Inc., 2020a) with seasonal S2 L2A reference images. For that, we performed cloud masking of the L2A data based on the scene classification and the cloud displacement index (Frantz et al., 2018) with a threshold of −0.8. We then created three seasonal near-infrared reference images, each covering a total of four months (September-December, January-April, May-August), to reduce seasonality effects in the coregistration procedure (Rufin et al., 2021a). We calculated displacement vectors for the PlanetScope near-infrared bands based on the S2 reference using the displacement function in Google Earth Engine. The algorithm performs a multi-scale rubber-sheeting correction based on cross-correlation. After several tests, we defined a maximum offset as 100 m and stiffness as 5 and used the resulting displacement vectors to coregister all PlanetScope spectral bands. We divided the study area into 0.3° grid tiles and processed each tile individually with a 0.05° buffer to avoid edge artifacts.

We assessed the effects of the coregistration procedure by calculating Normalized Difference Vegetation Index (NDVI) time series noise (Vermote et al., 2009) from triplets of measurements $y_{i}$, $y_{i+1}$, and $y_{i+2}$ acquired at ${\mathrm{month}}_{i}$, ${\mathrm{month}}_{i+1}$, and ${\mathrm{month}}_{i+2}$. We quantified the differences between the center NDVI and the linear interpolation between the two outer measurements as time series noise:

(1) $$\mathrm{TSNoise}=\sqrt{\frac{\sum_{i=1}^{n-2}{\left(y_{i+1}-\frac{y_{i+2}-y_{i}}{\mathrm{mont}h_{i+2}-month_{i}}\left(\mathrm{mont}h_{i+1}-month_{i}\right)-y_{i}\right)}^{2}}{N-2}}$$

We created spatial representations of time series noise for selected sites and visually inspected the geometric consistency of time series metrics across 10% of the tiles during training data collection to assess the effect of the coregistration procedure.

Based on the coregistered mosaics, we generated time series metrics for three seasons ranging from September through December, January through April, and May through August (Bey et al., 2020). For every season, we calculated median (P50) values for the green, red, and near-infrared band, as well as NDVI, for which we added a 75th percentile metric (P75). In addition, we included texture and contrast based on the first season and third season median NDVI, because previous studies have shown the value of textural information in classifying smallholder agriculture in the region (Bey et al., 2020). Texture indices (TI) were derived by calculating median values in 25 m, 100 m, and 200 m radial kernels, yielding three texture layers for two seasonal windows. The kernel sizes were determined by trial-and-error and are geared towards reflecting the distribution of field extents in the region. Contrast-enhancing indices (CI) were subsequently derived as normalized difference between pixel-level seasonal NDVI P50 and the corresponding TI:

(2) $${\mathrm{CI}}_{P50NDVI}=\frac{\left(P50_{\mathrm{NDVI}}-{\mathrm{TI}}_{NDVIP50}\right)}{\left(P50_{\mathrm{NDVI}}+{\mathrm{TI}}_{NDVIP50}\right)}$$

**Fig. 3:**
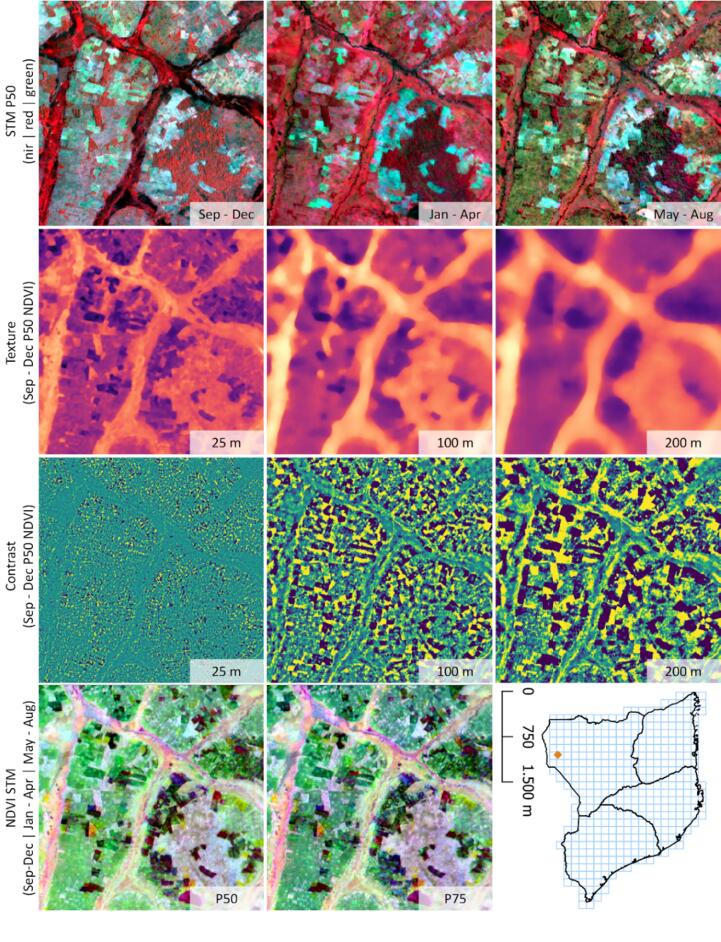
Input features derived from the PlanetScope mosaics. Rows show false-color infrared visualization of the seasonal median metrics, the texture of the third season NDVI median across different kernel sizes, contrast-enhancing features based on different texture kernels, and RGB composite of seasonal NDVI metrics.

These features normalize the NDVI values of a specific pixel with its surroundings, a technique that has been successful in large-area agriculture mapping studies (Deines et al., 2019). The texture and contrast metrics were designed to emphasize the contrasts between actively used fields and the surrounding landscape (Fig. 3). In addition, we included elevation, slope, latitude, and longitude as input layers for the classification, as these have been shown to improve classification performance in large-area mapping studies (Pflugmacher et al., 2019). We derived elevation and slope from the NASADEM (NASA JPL, 2020) digital elevation model and created layers representing the latitude and longitude of each pixel in geographic coordinates in Google Earth Engine.

### 2.4 Model training & classification

Active learning facilitates the selection of additional training locations in an interative fashion, e.g., through the use of model-based uncertainty estimates (Stumpf et al., 2014, Tuia et al., 2011). We used an active learning routine for iteratively amending our training dataset using Random Forest class probabilities.

We created an initial training dataset within a 10% random sample of the 422 0.3° tiles (n = 42). Within each tile, we randomly sampled 50 points with a minimum distance of 0.002° (∼222 m) for initial model training. For each point, we screened the seasonal metrics and Google Earth very high resolution (VHR) imagery to record the class label according to our classification scheme (Table 1) and the VHR acquisition date. If no appropriate label could be obtained for the target location, the sample was moved into a nearby region to determine a class label.

**Table 1:** Class catalog, definitions, and training sample size for both iterations.

Class	Definition	Final (Initial) training samples	Validation samples
Active cropland	Actively used cropland with signs of recent land management	677 (582)	325
Short-term fallow cropland	Fallow croplands with active use in or after 2015	301 (209)	232
Herbaceous vegetation	Natural grasslands and wetlands	370 (239)	245
Open woodland	Open woody canopy with 10–75% cover fraction	650 (532)	383
Closed woodland	Closed woody canopy with >75% cover fraction, including forestry plantations.	601 (514)	336
Unvegetated	Open soil, built-up, rock	226 (168)	213
Water	Perennial water bodies	53 (51)	206

The short-term fallow class was particularly difficult to train. We labeled a location as short-term fallow only if Google Earth VHR data revealed a recent fallow stage and previous cultivation in the last five years. Due to the high requirements for suitable pairs of VHR imagery, the fallow class was underrepresented in the initial random training dataset. We, therefore, trained an initial Random Forest model (Breiman, 2001) with 250 trees and predicted class probabilities across the training tiles. The probability layers were used in conjunction with Google Earth VHR data to amend the number of samples for the short-term fallow class from 66 to 209.

We performed an initial classification and then further enhanced the training dataset by sampling eight additional tiles (2%), selected to match the distribution of cropland fractions in our training tiles with that of the study region. We labeled 50 random points per tile. Next, we calculated probability margins M_prob_ at the pixel level as the difference between the probability of the most likely and the second most likely class. We calculated the class-wise 25th percentile of M_prob_ for all pixels belonging to the respective class as a threshold to identify pixels which were classified with relatively low confidence. We then sampled fifty locations per class in regions with M_prob_ scores below the 25th percentile, amending the initial sample by 350 locations. The final training dataset comprising 2,878 samples was used to train our final Random Forest model using 250 trees.

We deliberately designated one remote sensing expert with field experience as interpreter for both training and validation, to avoid misinterpretations by inexperienced interpreters and resulting inconsistencies in the reference data, which would require further interaction by experts. The resulting reference dataset is expected to be of highest consistency and quality, which is crucial for the classification of smallholder cropland using Random Forests (Estes et al., 2021).

### 2.5 Validation and unbiased area estimation

We created a stratified random sample to validate the map results and generate an unbiased estimation of class areas following recommended procedures (Olofsson et al., 2014). We calculated sample size (n = 1,875) targeting a standard error of the overall accuracy of 1% and assuming Useŕs accuracies of 0.75 for all classes (Cochran, 1977). We allocated 200 samples per class and the remainder (n = 475) according to class proportions. Each sampling unit (pixel) was labeled based on false-color infrared representations of the time series metrics at three zoom levels, Google Earth VHR images (for cropland and fallows we constrained the use to those with acquisition dates after 2016), and S2 NDVI time series profiles obtained using the Google Earth Engine TimeSeries Explorer plugin for QGIS (Rufin et al., 2021b). We could determine a class label for 89.8% of the samples. We generated a confusion matrix and derived area-adjusted overall accuracy, class-wise useŕs and produceŕs accuracy, 95% confidence intervals, and error-adjusted area estimates from the reference data, using the mapac package for R v0.11 (Pflugmacher, 2022).

### 2.6 Assessing cropland distribution and comparing land cover products

We assessed the spatial patterns of cropland by calculating the share of active cropland, short-term fallow, and total cropland (including both active and fallow cropland) per 0.1° grid cell. We then investigated the relationship between the share of short-term fallows and the share of total cropland at the 0.1° grid cell level, which we consider to be descriptive of agricultural consolidation. We further considered accessibility expressed in travel times (minutes) to the next city (Weiss et al., 2018).

For comparison, we compiled several global and regional land cover and cropland products, from which we calculated the extent and spatial distribution of cropland at the 0.1° grid cell level. Note that product-specific definitions of cropland cause differences in mapped cropland extent. We provide the class definitions here in order to inform readers on differences in cropland definitions (Table 2). The two regional land cover products included here are one map produced by the Fundo Nacional de Desenvolvimento Sustentável (FNDS, 2019), and a second map developed in the context of tree plantation monitoring (Bey and Meyfroidt, 2021). Importantly, both regional products were principally produced for mapping forests and tree plantations and thus have a generic cropland definition involving both active cropland and short-term fallows.

**Table 2:** Key characteristics of land cover products used for comparison in this study.

Name	Cropland definition	Scope	Res. (m)	Year	Reference
ESA WorldCover	Land covered with annual cropland that is sowed/planted and harvestable at least once within the 12 months after the sowing/planting date. The annual cropland produces an herbaceous cover and is sometimes combined with some tree or woody vegetation. Note that perennial woody crops will be classified as the appropriate tree cover or shrub land cover type.	Global Land Cover	10	2020	(Zanaga et al., 2021)
ESRI Land Cover	Human planted/plotted cereals, grasses, and crops not at tree height; examples: corn, wheat, soy, fallow plots of structured land.	Global Land Cover	10	2020	(Karra et al., 2021 - 2021)
MOD12Q1 V006	Cropland where at least 60% of the area is cultivated cropland, and mosaics of small-scale cultivation 40–60% with natural tree, shrub, or herbaceous vegetation.	Global Land Cover	500	2019	(Sulla-Menashe et al., 2019)
COPERNICUS Land Cover 100 m C3	Lands covered with temporary crops followed by harvest and a bare soil period (e.g., single and multiple cropping systems). Note that perennial woody crops will be classified as the appropriate forest or shrubland cover type.	Global Land Cover	100	2019	(Buchhorn et al., 2020)
GLAD Cropland	Land used for annual and perennial herbaceous crops for human consumption, forage (including hay), and biofuel. Perennial woody crops, permanent pastures, and shifting cultivation are excluded from the definition. The fallow length is limited to 4 years for the cropland class.	Global Cropland	30	2019	(Potapov et al., 2021)
GFSAD30	All cultivated plants harvested for food, feed, and fiber, including plantations (e.g., orchards, vineyards, coffee, tea, rubber), and fallow areas, but excluding pastures.	Global Cropland	30	2015	(Xiong et al., 2017)
FNDS Land Cover	Non-woody crops with at least 20 %of use and a minimum mapping unit of 1 ha.	National Land Cover	10 – 30 *m*	2016	(FNDS, 2019)
Bey and Meyfroidt (2021)	Cultivated landscapes including active and fallow cropland at the 30 m level.	National Land Cover	30	2017	(Bey and Meyfroidt, 2021)

## 3 Results

### 3.1 Pre-processing

**Fig. 4:**
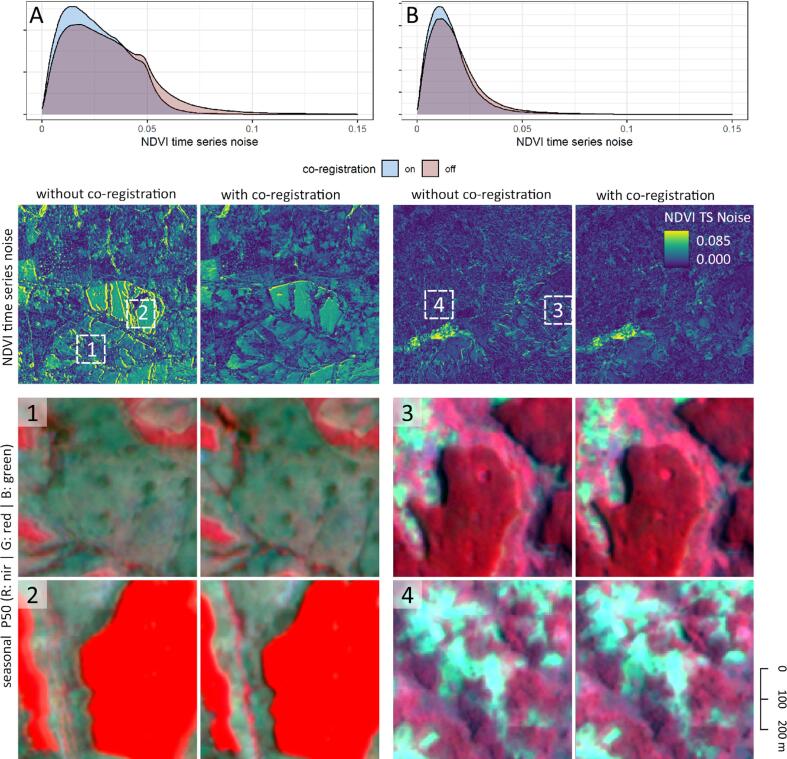
Time series noise as density and maps, with and without coregistration for two regions. Image chips represent subsets of seasonal median metrics within the presented regions.

Our geometric coregistration approach based on S2 reference images successfully mitigated multi-temporal coregistration issues in most cases (Fig. 4). The effect of the coregistration was most notable at sharp transitions of land cover classes, such as the edges of forestry plantations (Example A). More subtle effects were observed in fragmented landscapes (Example B). Importantly, high noise is not per se an indication of low geometric consistency but can also represent highly dynamic land surface characteristics, such as floodplains with multiple cropping cycles within a year. Remaining misregistration issues were observed in forested hillslopes and topographically challenging terrain, often in the seasonal window overlapping with the wet season. Aggregating the individual mosaics into seasonal median or 75th percentile metrics further eradicated mosaicking artifacts and cloud contamination. The code for creating the seasonal S2 reference images, coregistering the PlanetScope mosaics, and creating input metrics are available at https://github.com/philipperufin/eepypr/.

### 3.2 Accuracy assessment and area estimates

The area-adjusted overall accuracy of the final map was 88.6% (±1.5%), or 91.6% (±1.2%) when considering only the classes active cropland, fallow cropland, and non-cropland. Class-wise useŕs accuracies ranged between 71.1% (±4.9%) for the active cropland class to 99.0% (±1.3%) for water, produceŕs accuracies ranged between 61.4% (±5.8%) for herbaceous vegetation to 99.7% (±0.4%) for closed woodland. The iterative model training led to a 12% improvement in map accuracy as compared to the initial prediction (overall accuracy 76.0% (±2.1%)). The confusion matrix (Table 3) revealed that commission errors of the active cropland class were dominant, affecting herbaceous vegetation, open woodland, and short-term fallows. Omission errors of the active cropland class were negligible. Commission errors of short-term fallow occurred mostly on herbaceous vegetation, whereas omission errors mostly occurred in active cropland. Error-adjusted area estimates based on the reference sample revealed the overall class proportions and related confidence intervals. In the growing season 2020/2021, active cropland accounted for 16.2% (±1.1%), and fallow for 6.6% (±0.8%) of the study area. Area estimates for both cropland classes combined are 22.8% (±1.2%).

**Table 3:** Confusion matrix populated with probabilities, class-wise area-adjusted useŕs (UA) and produceŕs accuracy (PA) with 95% confidence intervals (CI), as well as sample-based area estimate with 95% confidence intervals (CI).

	Active Cropl.	Short Fallow	Herb. Veg.	Opn. Wdl.	Cld. Wdl.	Non-Veg.	Water	UA	95% CI.
Active Cropl.	0.1578	0.0116	0.0266	0.0205	0	0.0055	0	71.1%	4.9%
Short Fallow	0.002	0.0437	0.0035	0.0013	0	0	0	86.7%	4.4%
Herb. Veg.	0.0019	0.0058	0.0691	0.0006	0.0006	0.001	0	87.3%	4.2%
Opn Wdl.	0	0.0039	0.0097	0.3558	0	0.001	0	96.1%	1.9%
Cld Wdl.	0	0.0008	0.003	0.0129	0.2389	0	0	93.5%	2.6%
Non-Veg.	0.0002	0.0007	0.0004	0	0	0.0143	0.0003	89.7%	4.1%
Water	0	0	0.0001	0	0	0	0.0066	99.0%	1.3%
PA	97.5%	65.8%	61.4%	91.0%	99.7%	65.8%	95.6%		
95% CI	1.2%	7.3%	5.8%	2.2%	0.4%	13.2%	4.1%		
Sample-based area	16.2%	6.6%	11.2%	39.1%	24.0%	2.1%	0.7%		
95% CI	1.1%	0.8%	1.1%	1.2%	0.7%	0.4%	0.0%		

### 3.3 Spatial patterns of active and fallow cropland

**Fig. 5:**
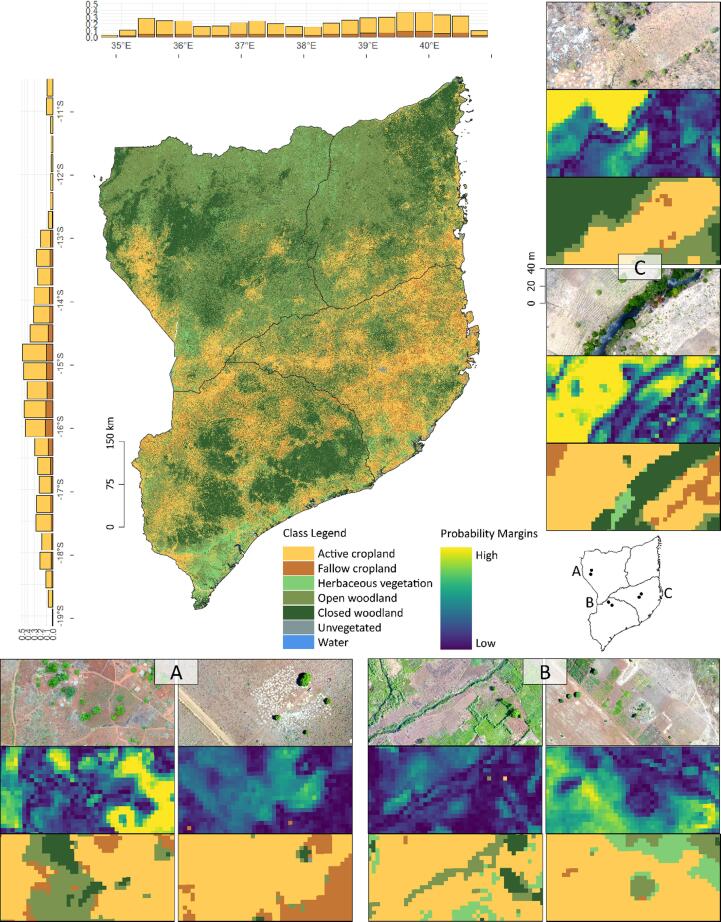
Map overview with histograms depicting cropland and fallow distribution by latitude and longitude. Zoom-ins show in-situ drone data obtained during fieldwork in mid-November 2021, probability margins, and classification outputs.

The province Nampula had the highest share of active cropland (40.3%), followed by Zambezia (26.8%), Cabo Delgado (15.4%), and Niassa (10.3%), with the Niassa reserve – a large protected area in the northeastern part of Niassa province and northwestern part of Cabo Delgado province – being essentially void of cropland (Fig. 5). The largest fractions of active cropland were mapped in the vicinity of larger agglomerations such as Mocuba and Pebane in Zambezia, Pemba in Cabo Delgado, Lichinga in Niassa province, and Nampula city in Nampula province. Besides these, the spatial patterns of active cropland follow the major transportation corridors along the coast and the east-west Nacala Corridor. The map is made available online for further use in compliance with the custom license of the NICFI data program (https://doi.org/10.5281/zenodo.6907606).

**Fig. 6:**
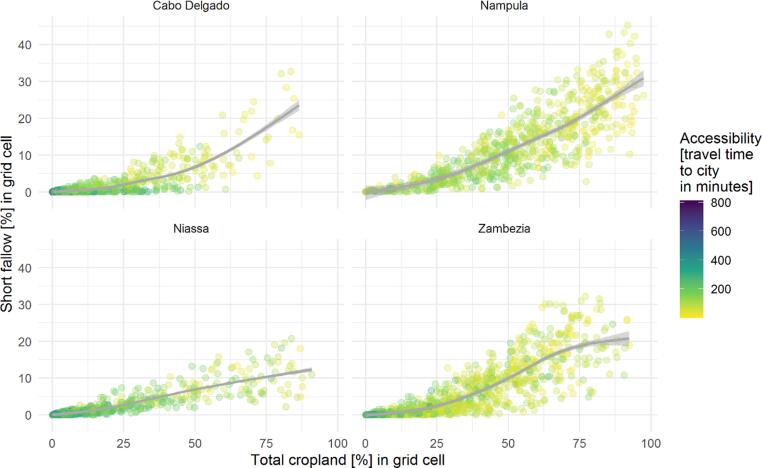
Relationship between total cropland (percentage per grid cell), short-term fallow (percentage per grid-cell), and accessibility (travel time to city in minutes) per 0.1° grid cell.

According to the maps, short-term fallows accounted for 13.2% of the total cropland area, though this remains an underestimate as our error-adjusted estimate indicates 28.9%. The most densely populated province Nampula had the highest shares of fallows (12.8%), followed by Zambezia (6.0%), Cabo Delgado (2.2%), and Niassa (1.4%). Fallow fractions (relative to total cropland) rarely exceeded 40% at the 0.1° grid level. Fallow fractions increased non-linearly with increasing cropland fractions and accessibility, indicating higher shares of short-term fallows in consolidated production regions (Fig. 6).

### 3.4 Comparing cropland extent across products

**Fig. 7:**
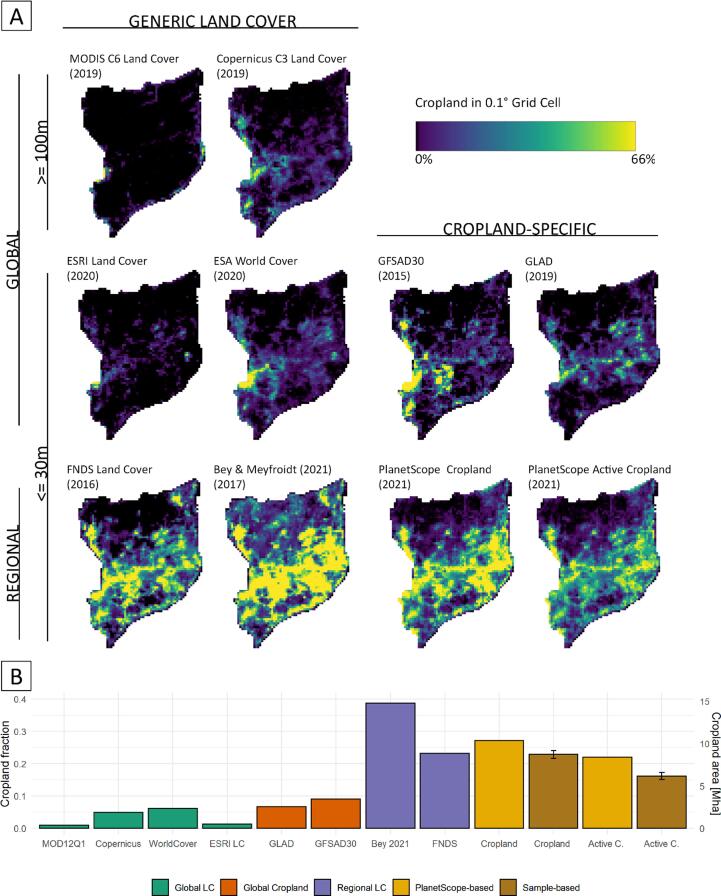
Cropland fractions for different land cover products (see Table 2 for details) per 0.1° grid cell (A) and aggregated for the entire study region (B).

A comparison at the grid-cell level revealed the similarities and differences in cropland distribution derived from the eight land cover products considered here (Fig. 7 A). Two of three global land cover products with a generic class catalog (ESRI Land Cover, MODIS Land Cover) show cropland fractions close to 1% in the study region (Fig. 7 B), and the WorldCover product indicated 6%. The cropland-specific products (GFSAD30, GLAD) showed shares of 7%, and 9%, respectively. The regional products show substantially higher shares of cropland, with 23% and 39%.

**Fig. 8:**
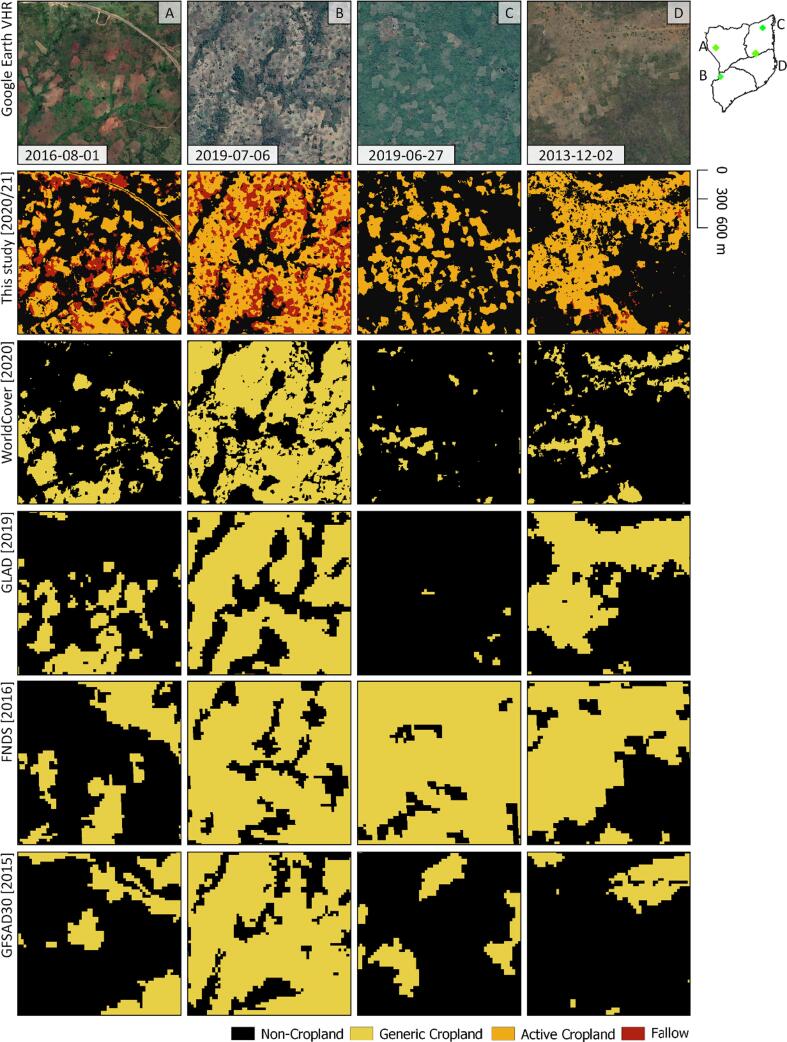
Image subsets of Google Earth VHR data, the PlanetScope based map, and selected land cover products, with target years in square brackets.

Snapshots of exemplary regions reveal the strengths and caveats of our PlanetScope-based maps in comparison with existing products mapping generic cropland (Fig. 8). First (A), the spatial detail of the PlanetScope data in conjunction with our pixel-based classification allows for the identification of very small fields, which may go unnoticed in other products. However, some commission errors are visible at land cover transitions, e.g. next to roads. Second (B), while existing products show good agreement in consolidated regions, only the PlanetScope-based map can be used to disentangle active from the fallow components of cropland. Third (C), our map accurately depicts active cropland in particularly dynamic landscapes, whereas large disagreement was found in existing products. Here, minimum mapping units (FNDS), object-based approaches (GFSAD30), multi-year aggregation of imagery, and cropland definitions involving fallows or excluding shifting cultivation (GLAD) may lead to highly differing representations of cropland extent. The omission of short-term fallows in the PlanetScope-based map here likely results from the high rates of vegetation growth, causing a high resemblance with open woodlands after short periods. Lastly (D), the PlanetScope-based map captures the landscape complexity, as it for instance includes small cropland parcels next to housing, and excludes trees on crop fields.

## 4 Discussion

This study presents a framework for mapping complex smallholder landscapes at 4.77 m resolution using PlanetScope data provided through the NICFI data program and Google Earth Engine. We combine state-of-the-art pre-processing techniques to create maps of active cropland and short-term fallows, besides five other land cover classes, for a large area in Northern Mozambique. Our maps are accurate and allow for assessments of the distribution of active cropland and short-term fallow cropland at unparalleled timeliness, spatial and thematic detail across more than 380,000 km^2^.

Our pre-processing framework for the PlanetScope mosaics produced consistent, spatially detailed, and temporally precise analysis-ready image features in a cloud-prone and topographically challenging region. The S2-based coregistration procedure in Google Earth Engine could not resolve all geometry-related artifacts, but nevertheless led to substantial improvements in the quality of time series metrics used for classification. The creation of seasonal median reflectance and index values further mitigated the presence of cloud and cloud shadow remnants. The iterative training procedure to amend our training dataset improved the classification substantially (+12.6% compared to the initial prediction). The inclusion of Random Forest probability layers was particularly valuable to identify training locations of short-term fallows, as its strict definition imposed high requirements on VHR data availability. This approach may be particularly useful for studies focusing on rare and hard-to-train classes, such as change classes, in the absence of reference data.

The resulting maps substantially improve on existing approaches in terms of spatial resolution and thematic detail, with accuracies in line with or exceeding those of previous approaches on active cropland mapping in complex smallholder regions (Estes et al., 2021, Ibrahim et al., 2021). Similar to previous works, commission errors of cropland were a key error type (Estes et al., 2021, Fnds, 2019, Xiong et al., 2017), which should be a focus of further research. We observed this error in land cover transition zones, such as between moist herbaceous vegetation and dry, bright sandy soils. Moreover, we observed the omission of fallows in regions with higher soil moisture due to confusion with herbaceous vegetation. Integrating information on moisture availability, e.g. through optical measurements in the shortwave infrared domain, could potentially mitigate these errors.

Comparing global and regional land cover products revealed a substantial variation in overall cropland extent. While mismatches between the target year of our study and those of existing products may contribute to the observed differences, the interplay of spatial resolution or minimum mapping units, production methods, spatial coverage, and, as a result, differing class definitions certainly play a key role in the observed differences. Generic cropland definitions (including fallow) are commonly used due to mixtures of non-crop vegetation, active and fallow cropland at the sub-pixel level. However, they limit the usefulness of products for estimating the area of active cropland and fallows, which are key for assessments of agricultural productivity, food security, livelihoods, and land change.

Knowledge about the fractions and spatial distribution of fallows in SSA is scarce. We found that short-term fallows occupy a substantial part of the cropland area, with a high spatial heterogeneity. Our short-term fallow fractions (map-based 13%, sample-based 29%) are in line with fallow fractions of 25% reported at the farm-level in other parts of Mozambique (Leonardo et al., 2018), whereas farm-level decisions may lead to varying fractions and lengths of fallows (Temudo and Silva, 2012). Large differences compared to a remote-sensing-based estimate documenting fallow fractions of 63% in the Sahel belt (Tong et al., 2020) can be explained by regional differences as well as through our narrow fallow definition, which excludes long-term fallows of more than 5 years of length. Additionally, our map entails omission errors of short-term fallows in regions with smaller agricultural footprints and high vegetation growth rates, where fallows were identified as woody cover. Integrating PlanetScope imagery from past growing seasons may help to delineate a broader range of fallow types and stages, including those with high fractions of woody cover. However, the reduced temporal density of the PlanetScope mosaics in the pre-2020 era and the limited availability of S2 L2A products in Google Earth Engine was a major constraint for including pre-2020 PlanetScope mosaics to broaden the definition of fallow land in this study. The continuation of the NICFI data program will allow for the enhancement of the methods presented here in terms of disentangling short-term and long-term fallows and providing detailed insights into year-to-year change processes.

## 5 Conclusion

Spatially detailed and timely maps on active cropland and short-term fallow in smallholder landscapes are pivotal for assessments of food security, livelihoods of local communities, land use change, and carbon budgeting, but are commonly not available. This work provides a framework for mapping active cropland and short-term fallows in highly fragmented smallholder landscapes. Our approach relies on PlanetScope mosaics made available through the NICFI data program. We derived seasonal analysis-ready datasets from coregistered time series of PlanetScope mosaics and used iterative learning to map active and fallow cropland using Google Earth Engine. The resulting map covers 380,000 km^2^ at <5 m spatial resolution, separates active cropland from short-term fallows in the growing season 2020/2021, and is highly accurate (88.6% ± 1.5%). The PlanetScope-based cropland map presented here enables more precise estimates of actively used cropland through its high granularity, temporal precision, and thematic depth untangling active cropland and short-term fallows. Our approach is operational and thus suitable to tackle persistent constraints related to spatial complexity and dynamics for mapping complex smallholder landscapes in the tropics.

### CRediT authorship contribution statement

**Philippe Rufin:** Conceptualization, Methodology, Formal analysis, Software, Writing – original draft, Writing – review & editing. **Adia Bey:** Conceptualization, Writing – review & editing. **Michelle Picoli:** Conceptualization, Writing – review & editing. **Patrick Meyfroidt:** Conceptualization, Writing – review & editing, Supervision, Funding acquisition.

## Declaration of Competing Interest

The authors declare that they have no known competing financial interests or personal relationships that could have appeared to influence the work reported in this paper.

## Data Availability

The data and code presented here is made available at https://doi.org/10.5281/zenodo.6907606 and https://github.com/philipperufin/eepypr/.
